# Assessment of Expected Out-of-Pocket Spending for Rheumatoid Arthritis Biologics Among Patients Enrolled in Medicare Part D, 2010-2019

**DOI:** 10.1001/jamanetworkopen.2020.3969

**Published:** 2020-04-27

**Authors:** Alexandra Erath, Stacie B. Dusetzina

**Affiliations:** 1 Vanderbilt University School of Medicine, Nashville, Tennessee; 2 Department of Health Policy, Vanderbilt University School of Medicine, Nashville, Tennessee

## Abstract

**Importance:**

The closure of the Medicare Part D coverage gap from 2010 to 2019 was intended to help decrease out-of-pocket costs for beneficiaries, especially those taking high-cost drugs. However, yearly increases in list prices and the introduction of newer and more expensive drugs may have limited savings for beneficiaries.

**Objective:**

To assess the association of closure in the Medicare Part D coverage gap with projected annual out-of-pocket costs from 2010 through 2019 for rheumatoid arthritis (RA) biologics.

**Design, Setting, and Participants:**

This cross-sectional analysis used data from the Medicare Formulary and Pricing Files for the first quarter (January 1 to March 31) in each calendar year from 2010 to 2019 for 17 RA biologic drug and strength combinations.

**Exposures:**

Medicare Part D plan design and drug price by year.

**Main Outcomes and Measures:**

Expected annual out-of-pocket costs for 1 year of treatment.

**Results:**

Among the 17 drug and strength combinations assessed, list prices increased each year for every product, with a mean increase of 160% for the 6 drugs available during the entire study period. For the 6 products available during the entire study period, projected mean (SD) annual out-of-pocket costs were 34% (2%) lower in 2011 than in 2010 ($6108 in 2010 to $4026 in 2011) but only 21% (8%) lower in 2019 ($4801) because of yearly increases in list price. All 4 products with higher out-of-pocket costs in 2019 than in the first year available entered the market between 2011 and 2015. For all products studied, the percentage of money spent in the catastrophic phase increased each year and was a mean (SD) of 22% (14%) higher in 2019 than in 2010 or the year first available.

**Conclusions and Relevance:**

Although beneficiaries experienced large reductions in out-of-pocket spending from 2010 to 2011, more than half of those savings were lost by 2019 because of annual increases in list prices, even as the coverage gap continued to close in subsequent years.

## Key Points

**Question:**

Was the closure of the coverage gap in Medicare Part D from 2010 to 2019 associated with decreased annual out-of-pocket costs for specialty rheumatoid arthritis drugs?

**Findings:**

In this cross-sectional study of 17 drug and strength combinations, projected annual mean out-of-pocket costs for rheumatoid arthritis treatments decreased 34% between 2010 and 2011 as the coverage gap began closing. By 2019, out-of-pocket spending was 21% lower than in 2010, suggesting that list price increases outpaced savings in subsequent years.

**Meaning:**

Although the projected annual out-of-pocket cost of many rheumatoid arthritis biologics was lower in 2019 than it was before the coverage gap closed, much of the cost savings of closing the gap was already lost to yearly price increases.

## Teaser

This cross-sectional study assesses the association between closure of the Medicare Part D coverage gap and projected annual out-of-pocket costs from 2010 through 2019 for rheumatoid arthritis biologics.

## Introduction

In the standard Medicare Part D benefit design, the coverage gap (ie, “donut hole”) has traditionally exposed patients to high out-of-pocket costs, which have been associated with cost-related medication nonadherence and unpredictable annual drug expenses.^1,2,3^ From 2006 through 2010, patients were responsible for 100% of drug costs while in the gap. In 2010, this situation required patients who reached the gap to spend $3610 out of pocket before moving into the catastrophic phase of the benefit (in which spending decreased from 100% to 5% of the drug’s list price). The landmark 2010 Patient Protection and Affordable Care Act (ACA) represented an overhaul of the US health care system and included a decade-long plan to close the coverage gap by introducing yearly reductions in patient out-of-pocket cost in the gap phase.^4^ These reductions were offset by requiring manufacturers to pay a 50% discount on brand-name drugs beginning in 2011 and by gradually increasing plan contributions (from 0% in 2011 to 25% in 2020). The Bipartisan Budget Act of 2018 increased manufacturer discounts from 50% to 70% and reduced plan payments to 5% for branded drugs filled in the coverage gap in 2019.^5^ With these changes, by 2019, Medicare Part D beneficiaries pay 25% of a brand-name drug’s list price in the coverage gap. Early research has found that in its first few years of implementation, the ACA was associated with decreased overall out-of-pocket spending on prescription drugs and that this association was most pronounced for those patients who enter the coverage gap.^1,6^

Despite improved financial protections for some patients, those with high drug spending remain at significant financial risk. Although their cost sharing in the gap has decreased since 2010, patients have remained responsible for 5% of the list price of filled drugs after they reach the out-of-pocket gap-phase threshold and enter the catastrophic phase of coverage. Over time, the number of patients reaching the catastrophic phase of the benefit has increased, as has their out-of-pocket spending.^7^ This situation has been associated with yearly increases in drug prices and the continued introduction of new and higher-cost specialty drugs over time.^8,9,10,11^

For patients, these expensive specialty drugs can represent a substantial ongoing financial liability because there is no limit on patient out-of-pocket spending in the catastrophic phase. In addition to financial hardships, increased cost exposure has important clinical implications associated with delayed initiation of treatment as well as higher rates of prescription abandonment.^12,13^ Prior work^14^ has found that price increases have erased anticipated savings for Medicare beneficiaries using anticancer treatments, but whether this phenomenon exists in other specialty drug markets is unclear.

Rheumatoid arthritis (RA) represents one of the largest markets for expensive specialty drugs^15^; moreover, patients with RA tend to take specialty medications for long periods because their disease is typically not life-shortening and requires consistent biologic therapy to remain controlled. For such patients, there remains a question regarding the effectiveness of the ACA’s changes to Medicare Part D standard benefit designs in limiting these patients’ annual out-of-pocket costs. Our objectives were to estimate expected out-of-pocket spending for Part D enrollees using biologic therapy for RA before and after the coverage gap closed, comparing 2010 data with 2019 data.

## Methods

This cross-sectional study used the Centers for Medicare & Medicaid Services Prescription Drug Plan Formulary, Pharmacy Network, and Pricing Information files quarterly data set for 2010 through 2019. This study was deemed exempt from review by the Vanderbilt University School of Medicine Institutional Review Board. Informed consent was waived because this research did not involve human participants. The study followed the Strengthening the Reporting of Observational Studies in Epidemiology (STROBE) reporting guideline.

The unit of observation was the Medicare Part D plan-year for each drug rather than individual beneficiary-level data. Quarter 1 (January 1 to March 31) data were used to reflect benefit designs for the entire year. To identify the drugs of interest, we conducted a literature search of the biologic medications currently approved and recommended to treat RA and that were primarily reimbursed through Part D (outpatient drug benefit).^16,17^ We limited the study to biologic medications because the analysis of interest centers on specialty drugs (which include biologics with high list prices). Included drugs were required to have entered the market by 2018 to allow comparison across years. National Drug Codes (NDCs) corresponding to a form of a drug expressly marketed for another indication (ie, Humira Crohn’s Disease Starter Pack) were excluded from our analysis because physicians are unlikely to prescribe these specific products for the treatment of RA (eTable 1 in the Supplement gives detailed information on product exclusions).

### Statistical Analysis

We calculated the median point-of-sale price per fill (typically representing a 30-day supply) for each drug in 2010 (or the year of market entry) through 2019, adjusted for medical inflation. Next, we examined coverage rates for each drug, defined as the percentage of formularies with the drug available and the percentage of these formularies that used a coinsurance (vs co-payments) for setting patient cost sharing and the median coinsurance used. When prescribing information indicated that 2 doses or forms of a drug were equivalent therapy choices, we also calculated coverage for that drug for all strengths and routes of administration. Next, we calculated annual projected out-of-pocket spending using the median coinsurance and the median point-of-sale price per fill, assuming patients fill 12 prescriptions per year and no other medications. All annual out-of-pocket spending was also inflation adjusted to 2019 dollars. For NDCs that corresponded to a quantity limit greater or less than the usual number of doses per month, we applied a multiplier to standardize costs for an expected 12 prescriptions. A complete list of the NDCs included and multipliers used is given in eTable 2 in the Supplement. Finally, we estimated costs by benefit phase for patients filling prescriptions in 2010 and those filling the same prescriptions in each year from 2011 through 2019 to determine how out-of-pocket spending changed overall and by coverage phase over time. In 2010, $250 was subtracted from the total out-of-pocket cost for the year to reflect the 1-time $250 check received by beneficiaries who reached the coverage gap as mandated by the ACA. Analyses were completed using Stata software, version 16.0.801 (StataCorp).

## Results

We investigated 17 drug and strength combinations. Table 1 lists the overview characteristics of the key drugs of interest. For all drugs studied, most plans used a coinsurance cost-sharing model in the initial coverage phase. After adjusting all prices to 2019 US dollars, the median price per fill increased for all drugs studied. Prices increased by more than 20% for every drug that had been on the market for more than 5 years, with the exception of the 100-mg/1-mL golimumab autoinjector, which entered the market in 2015 at a higher price than its existing formulation ($3867 vs $3306 for the 50-mg/0.5-mL golimumab autoinjector). The largest price increase was for the 20-mg/0.4-mL formulation of adalimumab, which increased from $1894 per fill in 2010 to $5299 per fill in 2019. For the 6 products on the market since 2010, the median list price increased a mean (SD) of 160% (17%; range, 136%-180%) by 2019.

**Table 1.** Baseline Characteristics and Median List Price, Percentage of Coverage, and Coinsurance Information for Drugs of Interest

Product	First available year (2010-2019)	Median list price per fill, $^a^		Coverage, %			
		First available year	2019	2010 or year of market entry	Part D plans covering product in 2019	Part D plans requiring coinsurance	
						2010 or year of market entry	2019
Tocilizumab, 162 mg/0.9 mL	2015	796	1002	37	37	98	100
Certolizumab pegol							
200 mg	2010	1795	4400	67	26	98	100
200 mg/1 mL	2011	1885	4343	64	26	97	100
Etanercept							
25 mg	2010	1007	2628	85	73	98	100
50 mg/1 mL	2010	2019	5253	85	73	98	100
Adalimumab							
10 mg/0.2 mL	2016	4216	5309	99	100	100	100
20 mg/0.4 mL	2010	1895	5300	45	100	98	100
40 mg/0.8 mL	2010	1890	5254	100	100	98	100
Sarilumab							
150 mg/1.14 mL	2018	3157	3385	7	30	100	100
200 mg/1.14 mL	2018	3157	3385	7	30	100	100
Abatacept							
125 mg/1 mL	2012	2307	4347	52	39	99	100
50 mg/0.4 mL	2018	4359	4442	42	39	100	100
87.5 mg/0.7 mL	2018	4359	4442	42	39	100	100
Golimumab							
100 mg/1 mL	2015	3867	5658	50	30	100	100
50 mg/0.5 mL	2010	2065	4873	51	30	98	100
Tofacitinib							
5 mg	2014	2510	4528	39	66	100	100
Extended-release 11 mg	2017	4101	4536	60	62	100	100

^a^ All prices were inflation adjusted and are reported in 2019 dollars.

**Figure 1.**
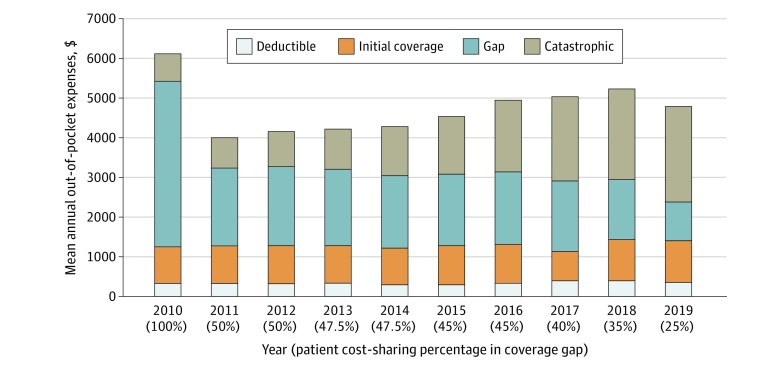
Mean Annual Out-of-Pocket Expenses Across All Products Available in 2010 to 2019 by Phase of Spending The 6 products available from 2010 through 2019 included in this mean analysis were 200 mg of certolizumab pegol, 25 mg of etanercept, 50 mg of etanercept, 20 mg/0.4 mL of adalimumab, 40 mg/0.8 mL of adalimumab, and 50 mg/0.5 mL of golimumab. Note that the annual out-of-pocket cost in 2010 does not reflect the 1-time $250 check given to patients who reached the gap in that year.

**Figure 2.**
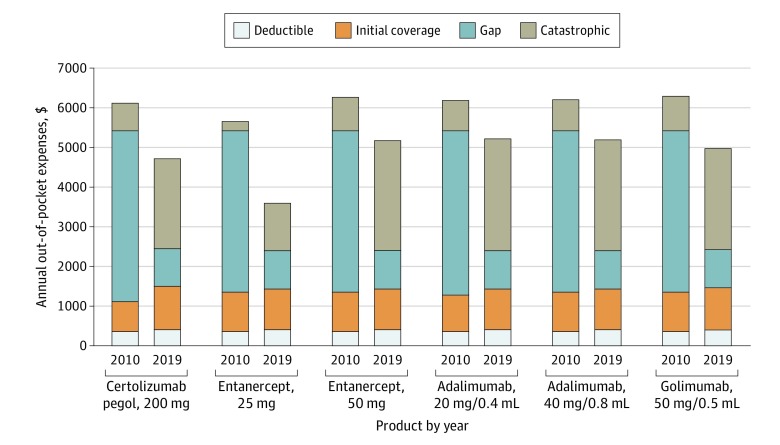
Annual Out-of-Pocket Expenses in 2010 vs 2019 by Phase of Spending for Products Entering the Market Before 2010

For these same 6 drugs, Figure 1 shows the annual out-of-pocket amount spent per year averaged across all products, segmented by dollars spent in each phase of coverage. For the products available from 2010 through 2019, mean (SD) annual out-of-pocket spending decreased from $6108 ($234; (range, $5647-6282; $5858 after the 1-time $250 rebate) in 2010 to $4801 ($620; range, $3594-$5196) in 2019. However, these savings were largely attributable to the mandatory manufacturer 50% rebate for brand-name drugs filled in the coverage gap, which reduced patients’ cost exposure from 100% to 50% between 2010 and 2011. Despite continued reductions in required patient cost sharing as the coverage gap closed over time, there was a mean (SD) 19% (9%; range, 2%-26%) increase in the mean annual out-of-pocket cost from 2011 to 2019 (from $4026 to $4801). There was a slight decrease in estimated annual out-of-pocket spending from 2018 to 2019 as the Bipartisan Budget Act accelerated the closing of the coverage gap. This expedited closure resulted in a 10% decrease in gap cost exposure between 2018 and 2019 (vs the 2.5% and 5% decreases in the previous 8 years) and a 20% increase in manufacturer discount in the gap, which did not affect patient out-of-pocket costs directly but rather propelled patients into the catastrophic phase more quickly. As expected, gap spending decreased a mean (SD) of 46% (2%; range, 45%-50%) and catastrophic spending increased a mean (SD) of 38% (5%; range, 29%-41%) for the 6 products on the market from 2010 through 2019. Figure 2 shows the out-of-pocket spending in 2010 vs 2019 for these products.

For 13 of the 17 drug-strength combinations covered by Part D during the study period, expected annual out-of-pocket cost was lower in 2019 than in the first year the product was available (Table 2). Expected out-of-pocket spending was higher in 2019 for 4 of the 5 products entering the market from 2011 to 2015: 200 mg/1 mL of certolizumab pegol, 125 mg/mL of abatacept, 100 mg/1 mL of golimumab, and 5 mg of tofacitinib. For all 17 drugs, projected annual out-of-pocket cost was a mean (SD) of $4613 ($698; range, $2618-$5439) in 2019; the overall cost of tocilizumab was the lowest at $2618, and the next lowest was 25 g of etanercept at $3594. The 100-mg/1-mL dose of golimumab was the most expensive at a projected annual cost of $5439. Catastrophic spending increased a mean (SD) of 13% (14%) for products entering after 2010 and a mean (SD) of 8% (3%) for products entering after 2015.

**Table 2.** Projected Annual Out-of-Pocket Expenses in First Available Year vs 2019

Product	First available year (2010-2019)	Projected annual out-of-pocket cost, $		Change in annual out-of-pocket costs from first available year to 2019, $
		First available year, 2010-2018	2019	
Certolizumab pegol, 200 mg	2010	6108	4710	−1398
Etanercept				
25 mg	2010	5647	3593	−2054
50 mg/1 mL	2010	6254	5168	−1086
Adalimumab				
20 mg/0.4 mL	2010	6177	5196	−981
40 mg/0.8 mL	2010	6177	5168	−1009
Golimumab, 50 mg/0.5 mL	2010	6282	4967	−1315
Certolizumab pegol, 200 mg/1 mL	2011	4046	4676	630
Abatacept, 125 mg/1 mL	2012	4317	4625	308
Tofacitinib, 5 mg	2014	4218	4787	569
Tocilizumab, 162 mg/0.9 mL	2015	3225	2618	−607
Golimumab, 100 mg/1 mL	2015	5067	5439	372
Adalimumab, 10 mg/0.2 mL	2016	5290	5202	−88
Tofacitinib, extended-release 11 mg	2017	5025	4765	−260
Sarilumab				
150 mg/1.14 mL	2018	4524	4075	−449
200 mg/1.14 mL	2018	4524	4075	−449
Abatacept				
50 mg/0.4 mL	2018	5179	4682	−497
87.5 mg/0.7 mL	2018	5179	4682	−497

## Discussion

On average, annual out-of-pocket costs for patients taking specialty biologics for RA decreased from 2010 to 2019 as the coverage gap was closed, and for 14 of the 17 drugs studied, patients paid a lower annual out-of-pocket cost in 2019 than in 2010 or the year first available. However, these decreases were associated with a 50% decrease in gap cost exposure from 2010 to 2011, a large 1-time decrease masking underlying trends in the 9 years since. The nearly 20% increase in projected annual out-of-pocket cost from 2011 through 2019 for the 6 available products suggests that the small annual decreases (2.5%-10%) in patient cost exposure in the gap from 2011 through 2019 have not been sufficient to keep pace with yearly increases in list prices as well as the introduction of increasingly expensive drugs. Without the large decrease in patient coverage gap cost sharing from 2010 to 2011, the data from the past decade revealed that 4 of the 5 drugs entering the market between 2011 and 2015 had higher annual out-of-pocket costs in 2019 than in their year of entry. Of importance, these products became available after the 2010 to 2011 coverage gap change but have also been on the market for at least 5 years, which is long enough for the compounded associations of annual increases in list prices with further reductions in gap cost sharing. The sole exception to this trend was tocilizumab, which was also the lowest-cost product at $3225 in 2015 and $2618 in 2019. The lower price of tocilizumab was likely attributable to its mechanism of action as an interleukin inhibitor because current practice guidelines recommend a different drug class, a tumor necrosis factor inhibitor, as first-line biologic therapy.^16^

Although patients paid less in 2019 than they did in 2010, the question remains whether a decrease from $5858 in 2010 (after the $250 rebate check) to $4801 in 2019 accomplished the ACA’s goal of significantly easing the burden of prescription drug costs, especially as upward price trends in recent years have edged patients even closer to their out-of-pocket costs a decade ago. These prices continue to represent a substantial cost burden for older patients with RA and have important clinical implications. For Medicare patients, having RA is associated with a 3-fold increase in risk of cost-related treatment nonadherence,^18^ with research showing that patients with RA with the highest levels of cost exposure are almost 30 times more likely to abandon their initial prescription.^19^ In addition, the proportion of patient spending that occurs in the catastrophic phase has increased each year. Now that the coverage gap has been officially closed, annual increases in list prices of these drugs may further increase catastrophic-phase spending. The positive association between length of time on the market and mean increase in catastrophic spending, including a 38% increase for products available in 2010, 13% increase for products entering after 2010, and 8% increase for products entering after 2015, supports the possibility of continued growth in catastrophic phase spending. Given the current lack of an out-of-pocket maximum on Medicare Part D, this finding of increasing catastrophic spending can represent an enormous financial liability for patients. Finally, it is not uncommon for patients to switch between biologics because of inadequate clinical response or adverse events, with 1 study^20^ of Medicare patients estimating an approximately 10% switch rate in the first year of therapy alone. In contrast to the treatment algorithms available for many common diseases, the trial-and-error approach often used for patients with RA in whom the first choice biologic fails may be associated with an increased likelihood of patients being switched to new and more costly drugs because there is no guideline for sequential biologic treatments.

### Limitations

This study has limitations. It is limited by the use of list prices, which fail to capture the rebates that may reduce prices paid by Medicare Part D plans for drugs filled. However, patient out-of-pocket spending is based on list prices rather than net prices, limiting the effect on our analysis.^21^ To allow for year-to-year comparisons, we focused on the annual expense associated with a single biologic filled every month, ignoring other treatments filled under Medicare Part D. Most patients receive several other medications as well, which would result in our underestimating out-of-pocket spending by patients. In addition, this analysis focused on beneficiaries who did not qualify for a low-income subsidy because their cost sharing was fixed and would change only slightly during the study period.^22^ However, for the more than two-thirds of Medicare Part D patients who do not qualify for such assistance, there was a trend in increasing cost exposure during the last 9 years, and there is no longer a yearly decrease in patient cost sharing in the gap. Although not all patients with RA initiate biologic therapy, estimates for biologic therapy initiation in the first few years after diagnosis range from 20% to 40%.^23,24^ However, there has been a recent push toward earlier use of biologics to capitalize on a window of opportunity early in the disease course,^25^ suggesting that the percentage of patients with RA affected by high biologic costs will continue to increase. Of note, the patients with the highest disease burden will be most exposed to these costs.

In addition, it is possible that the continuing development of the RA biosimilar market will increase price competition for these therapies and make available additional treatment options at a lower cost. However, meaningful cost reduction from biosimilars is currently limited by aggressive litigation by the biologic manufacturers and an insufficient number of competitors to markedly affect price.^26^ In addition, a 2019 survey of US rheumatologists found that practitioners are hesitant to switch a patient with stable RA from a biologic to its biosimilar, indicating a substantial first-mover advantage for the biologics; that is, the shift from biologic to biosimilar may be more gradual if primarily occurring in biologic-naive patients with newly diagnosed RA.^27^

## Conclusions

The mean projected annual out-of-pocket cost for an RA biologic was lower in 2019 than in 2010 or year of first entry, and the closure of the coverage gap was associated with a benefit to consumers overall. However, from 2011 to 2018, mean annual cost exposure for an RA biologic increased over inflation every year, even with the benefit of yearly reductions in gap cost exposure. As the coverage gap is now considered closed, our results suggest a need for out-of-pocket maximums in the catastrophic phase to limit older Americans’ yearly financial burden and allow them to better estimate their annual drug costs. In the interim, however, limiting the allowed annual increase in list prices and capping out-of-pocket costs for Medicare Part D enrollees may be associated with decreased financial burden for patients receiving biologic therapies.

## Notes

Supplement. **eTable 1.** RA Specialty Drugs Excluded From Analysis and Reason for Exclusion **eTable 2.** NDC Codes Included in Analysis and Multiplier Applied

## References

[1] Bonakdar Tehrani A, Cunningham PJ Closing the Medicare doughnut hole: changes in prescription drug utilization and out-of-pocket spending among Medicare beneficiaries with Part D coverage after the Affordable Care Act. Med Care. 2017;55(1):-. doi:10.1097/MLR.0000000000000613 27547949

[2] Park YJ, Martin EG Medicare Part D’s effects on drug utilization and out-of-pocket costs: a systematic review. Health Serv Res. 2017;52(5):1685-1728. doi:10.1111/1475-6773.12534 27480577 PMC5583296

[3] Polinski JM, Kilabuk E, Schneeweiss S, Brennan T, Shrank WH Changes in drug use and out-of-pocket costs associated with Medicare Part D implementation: a systematic review. J Am Geriatr Soc. 2010;58(9):1764-1779. doi:10.1111/j.1532-5415.2010.03025.x 20863336 PMC2946375

[4] Patient Protection and Affordable Care Act, Pub L No. 111-148, 124 Stat 119 (2010).

[5] Bipartisan Budget Act of 2018. Pub L No. 115-123, 42 USC 1305 (2018).

[6] Cubanski J, Neuman T, Damico A Closing the Medicare Part D Coverage Gap: Trends, Recent Changes, and What’s Ahead. Kaiser Family Foundation; 2018.

[7] Cubanski J, Neuman T, Orgera J, Damico A No Limit: Medicare Part D Enrollees Exposed to High Out-of-Pocket Drug Costs Without a Hard Cap on Spending. Kaiser Family Foundation; 2017.

[8] Trish E, Xu J, Joyce G Growing number of unsubsidized Part D beneficiaries with catastrophic spending suggests need for an out-of-pocket cap. Health Aff (Millwood). 2018;37(7):1048-1056. doi:10.1377/hlthaff.2018.0006 29985706 PMC7268910

[9] Trish E, Xu J, Joyce G Medicare beneficiaries face growing out-of-pocket burden for specialty drugs while in catastrophic coverage phase. Health Aff (Millwood). 2016;35(9):1564-1571. doi:10.1377/hlthaff.2016.0418 27605634 PMC5573178

[10] Levinson D High-Price Drugs Are Increasing Federal Payments for Medicare Part D Catastrophic Coverage. US Dept of Health and Human Services, Office of Inspector General; 2017.

[11] Wineinger NE, Zhang Y, Topol EJ Trends in prices of popular brand-name prescription drugs in the United States. JAMA Netw Open. 2019;2(5):e194791. doi:10.1001/jamanetworkopen.2019.4791 31150077 PMC6547085

[12] Zhang Y, Baik SH, Zhou L, Reynolds CF, Lave JR Effects of Medicare Part D coverage gap on medication and medical treatment among elderly beneficiaries with depression. Arch Gen Psychiatry. 2012;69(7):672-679. doi:10.1001/archgenpsychiatry.2011.1402 22752233 PMC3390754

[13] Doshi JA, Li P, Huo H, Pettit AR, Armstrong KA Association of patient out-of-pocket costs with prescription abandonment and delay in fills of novel oral anticancer agents. J Clin Oncol. 2018;36(5):476-482. doi:10.1200/JCO.2017.74.5091 29261440

[14] Dusetzina SB, Huskamp HA, Keating NL Specialty drug pricing and out-of-pocket spending on orally administered anticancer drugs in Medicare Part D, 2010 to 2019. JAMA. 2019;321(20):2025-2027. doi:10.1001/jama.2019.4492 31135837 PMC6547115

[15] Lotvin AM, Shrank WH, Singh SC, Falit BP, Brennan TA Specialty medications: traditional and novel tools can address rising spending on these costly drugs. Health Aff (Millwood). 2014;33(10):1736-1744. doi:10.1377/hlthaff.2014.0511 25288417

[16] Moreland LM, Cannella A General principles of management of rheumatoid arthritis in adults. Accessed November 12, 2019. https://www.uptodate.com/contents/general-principles-of-management-of-rheumatoid-arthritis-in-adults

[17] Thaler K, Chandiramani DV, Hansen RA, Gartlehner G Efficacy and safety of anakinra for the treatment of rheumatoid arthritis: an update of the Oregon Drug Effectiveness Review Project. Biologics. 2009;3:485-498. doi:10.2147/BTT.S3579 20054439 PMC2802074

[18] Harrold LR, Briesacher BA, Peterson D, Cost-related medication nonadherence in older patients with rheumatoid arthritis. J Rheumatol. 2013;40(2):137-143. doi:10.3899/jrheum.120441 23322458 PMC3815617

[19] Hopson S, Saverno K, Liu LZ, Impact of out-of-pocket costs on prescription fills among new initiators of biologic therapies for rheumatoid arthritis. J Manag Care Spec Pharm. 2016;22(2):122-130. doi:10.18553/jmcp.2016.14261 27015251 PMC10397931

[20] Rosenblatt LLF, Cockrum P, Biologic switching rates among patients with rheumatoid arthritis. Paper presented at: 2013 ACR/ARHP Annual Meeting; October 29, 2013; San Diego, California.

[21] Dusetzina SB, Conti RM, Yu NL, Bach PB Association of prescription drug price rebates in Medicare Part D with patient out-of-pocket and federal spending. JAMA Intern Med. 2017;177(8):1185-1188. doi:10.1001/jamainternmed.2017.1885 28558108 PMC5722464

[22] Cubanksi J, Damico A, Neuman T 10 Things to know about Medicare Part D coverage and costs in 2019. Accessed November 12, 2019. https://www.kff.org/medicare/issue-brief/10-things-to-know-about-medicare-part-d-coverage-and-costs-in-2019/

[23] DeWitt EM, Lin L, Glick HA, Anstrom KJ, Schulman KA, Reed SD Pattern and predictors of the initiation of biologic agents for the treatment of rheumatoid arthritis in the United States: an analysis using a large observational data bank. Clin Ther. 2009;31(8):1871-1880. doi:10.1016/j.clinthera.2009.08.020 19808146 PMC3518838

[24] George MD, Sauer BC, Teng C, Biologic and glucocorticoid use after methotrexate initiation in patients with rheumatoid arthritis. J Rheumatol. 2018;47(2):343-350. 30275262 10.3899/jrheum.180178 PMC6443489

[25] Resman-Targoff BH, Cicero MP Aggressive treatment of early rheumatoid arthritis: recognizing the window of opportunity and treating to target goals. Am J Manag Care. 2010;16(9)(suppl):S249-S258. 21517638

[26] Yazdany J, Dudley RA, Lin GA, Chen R, Tseng CW Out-of-pocket costs for infliximab and its biosimilar for rheumatoid arthritis under Medicare Part D. JAMA. 2018;320(9):931-933. doi:10.1001/jama.2018.7316 30193264 PMC6142992

[27] Gibofsy A, Badawi SUS Rheumatologists’ beliefs and knowledge about biosimiliars: an ongoing survey. Paper presented at: 2019 ACR/ARP Annual Meeting; 2019; Atlanta, Georgia.

