# Supplementary material for: Assessment of Expected Out-of-Pocket Spending for Rheumatoid Arthritis Biologics Among Patients Enrolled in Medicare Part D, 2010-2019
Source: JAMA Netw Open. 2020 Apr 27;3(4):e203969. doi: 10.1001/jamanetworkopen.2020.3969 (PMC7186858; doi:10.1001/jamanetworkopen.2020.3969)
Supplement: Supplement. — eTable 1. RA Specialty Drugs Excluded From Analysis and Reason for Exclusion eTable 2. NDC Codes Included in Analysis and Multiplier Applied [file jamanetwopen-3-e203969-s001.pdf]

Supplementary Online Content

Erath A, Dusetzina SB. Assessment of expected out-of-pocket spending for rheumatoid arthritis biologics among patients enrolled in Medicare Part D, 2010-2019. *JAMA Netw Open*. 2020;3(4):e203969. doi:10.1001/jamanetworkopen.2020.3969

eTable 1. RA Specialty Drugs Excluded From Analysis and Reason for Exclusion

eTable 2. NDC Codes Included in Analysis and Multiplier Applied

This supplementary material has been provided by the authors to give readers additional information about their work.

© 2020 Erath A et al. *JAMA Network Open.*

eTable 1. RA Specialty Drugs Excluded From Analysis and Reason for Exclusion

Product Name	Reason for Exclusion
Inflectra 100 mg	Biosimiliar; analysis limited to branded medications
Kineret 100 mg/0.67 mL	Third-line RA medication shown to be inferior to other biologic agents and excluded from other analyses for low market share[17, 22]
Remicade 100 mg	Investigation of Medicare spending dashboards found that the majority of spend for this drug is adjudicated through Part B rather than Part D.
Rituxan 10 mg/1 mL	
Simponi Aria 50 mg/0.4 mL	

© 2020 Erath A et al. *JAMA Network Open.*

eTable 2. NDC Codes Included in Analysis and Multiplier Applied

Product Name and Strength	NDC Codes	Multiplier
Actemra 162 mg/0.9 mL	50242013801	1
Cimzia 200 mg	50474070062	1
Cimzia 200 mg/1 mL	50474071079	1
Enbrel 25 mg	58406042534<br>58406042541	1
Enbrel 50 mg/1 mL	58406043501<br>58406044501	4
	58406045504	2
	58406043504<br>58406044504	1
Humira 10 mg/0.2 mL	00074634702	1
Humira 20 mg/0.4 mL	00074937402	1
Humira 40 mg/0.8 mL	00074379902<br>00074433902	1
Kevzara 150 mg/1.14 mL	00024590801	1
Kevzara 200 mg/1.14 mL	00024591001	1
Orencia 125 mg/1 mL	00003218811<br>00003218831	1
Orencia 50 mg/0.4 mL	00003281411	1
Orencia 87.5 mg/0.7 mL	00003281811	1
Simponi 100 mg/1 mL	57894007101<br>57894007102	1
Simponi 50 mg/0.5 mL	57894007001<br>57894007002	1
Xeljanz 5 mg	00069100101	1
Xeljanz XR 11 mg	00069050130	1

© 2020 Erath A et al. *JAMA Network Open.*
